# Standard and competing risk analysis of the effect of albuminuria on cardiovascular and cancer mortality in patients with type 2 diabetes mellitus

**DOI:** 10.1186/s41512-018-0035-4

**Published:** 2018-07-23

**Authors:** Benjamin G. Feakins, Emily C. McFadden, Andrew J. Farmer, Richard J. Stevens

**Affiliations:** Nuffield Department of Primary Care Health Sciences, University of Oxford, Radcliffe Observatory Quarter, Woodstock Road, Radcliffe Primary Care Building, Oxford, Oxfordshire OX2 6GG UK

**Keywords:** Competing risks, Survival analysis, Albuminuria, Type 2 diabetes mellitus, Cardiovascular mortality, Cancer mortality

## Abstract

**Background:**

Competing risks occur when populations may experience outcomes that either preclude or alter the probability of experiencing the main study outcome(s). Many standard survival analysis methods do not account for competing risks. We used mortality risk in people with diabetes with and without albuminuria as a case study to investigate the impact of competing risks on measures of absolute and relative risk.

**Methods:**

A population with type 2 diabetes was identified in Clinical Practice Research Datalink as part of a historical cohort study. Patients were followed for up to 9 years. To quantify differences in absolute risk estimates of cardiovascular and cancer, mortality standard (Kaplan-Meier) estimates were compared to competing-risks-adjusted (cumulative incidence competing risk) estimates. To quantify differences in measures of association, regression coefficients for the effect of albuminuria on the relative hazard of each outcome were compared between standard cause-specific hazard (CSH) models (Cox proportional hazards regression) and two competing risk models: the unstratified Lunn-McNeil model, which estimates CSH, and the Fine-Gray model, which estimates subdistribution hazard (SDH).

**Results:**

In patients with normoalbuminuria, standard and competing-risks-adjusted estimates for cardiovascular mortality were 11.1% (95% confidence interval (CI) 10.8–11.5%) and 10.2% (95% CI 9.9–10.5%), respectively. For cancer mortality, these figures were 8.0% (95% CI 7.7–8.3%) and 7.2% (95% CI 6.9–7.5%). In patients with albuminuria, standard and competing-risks-adjusted estimates for cardiovascular mortality were 21.8% (95% CI 20.9–22.7%) and 18.5% (95% CI 17.8–19.3%), respectively. For cancer mortality, these figures were 10.7% (95% CI 10.0–11.5%) and 8.6% (8.1–9.2%). For the effect of albuminuria on cardiovascular mortality, regression coefficient values from multivariable standard CSH, competing risks CSH, and competing risks SDH models were 0.557 (95% CI 0.491–0.623), 0.561 (95% CI 0.494–0.628), and 0.456 (95% CI 0.389–0.523), respectively. For the effect of albuminuria on cancer mortality, these values were 0.237 (95% CI 0.148–0.326), 0.244 (95% CI 0.154–0.333), and 0.102 (95% CI 0.012–0.192), respectively.

**Conclusions:**

Studies of absolute risk should use methods that adjust for competing risks to avoid over-stating risk, such as the CICR estimator. Studies of relative risk should consider carefully which measure of association is most appropriate for the research question.

**Electronic supplementary material:**

The online version of this article (10.1186/s41512-018-0035-4) contains supplementary material, which is available to authorized users.

## Background

### Statistical background

In prognostic studies, time-to-event data may be incomplete for many subjects as a result of loss to follow-up, reaching the end of the study, or withdrawal from the study. The truncation of subject follow-up resulting from incomplete data is typically referred to as ‘censoring’. Most standard survival models, like the Kaplan-Meier estimator [1] and Cox proportional hazards (Cox-PH) regression [2], assume that censoring is ‘non-informative’, meaning that a subject censored at a certain time point should be representative of those still under observation at the same time point [3], i.e. subject censoring times and event times should be independent. In practice, when studying any one cause of mortality, the censoring that can occur through death from other causes will rarely be non-informative.

Patients are seldom at risk of experiencing only a single type of outcome.

Competing risks are defined as events during follow-up that either preclude the observation of the primary outcome, or alter the probability of its occurrence [4]. The effect of competing risks was first acknowledged by d’Alembert and Bernoulli in the 1760s in relation to the effects of inoculation on short- and long-term mortality from smallpox [5]. The initial deterministic model of Bernoulli described two disease states (a ‘susceptible’ state and an ‘immune’ state), in addition to the absorbding state of ‘death’. In this model, subjects could transition from the susceptible state to the immune state at a rate dependent upon the force of infection, from either disease state to the state of death at the background death rate, and from the susceptible state to the state of death at an additional rate that was also dependent upon the force of infection. Bernoulli assumed no intermediate ‘infected’ disease state as smallpox infection was typically brief in duration (weeks) when compared to the other disease states. d’Alembert produced an alternative solution, which was more generalizable in its application as it was not restricted to immunising diseases. In this approach, d’Alembert described a single state of being ‘alive’ and two separate absorbing states of ‘death’, resulting from either the disease or all other causes. The rates of transition to each absorbing state were assumed by d’Alembert to be independent. These early disparities in approach continue to be born out to this day with study authors being able to choose from multiple methods to account for competing risks in studies. In this paper, we consider two standard survival analysis methods [1, 2] and compare the results to those obtained via three survival analysis methods adjusted for the presence of competing risks [4, 6, 7].

### Clinical background

Roughly 3.5 million people are currently living with diabetes mellitus in the UK, of which 90% have type 2 diabetes mellitus (T2DM) [8]. T2DM is a metabolic disorder that is characterised by defects in insulin sensitivity or secretion [9]. The disease typically manifests in later life and is progressive in nature [10], resulting in systemic macro- and microvascular complications [11]. One such complication is diabetic nephropathy whereby damage to the microvasculature of the glomerular capillaries of the kidneys results in a leakage of proteins into the urine (albuminuria/proteinuria), hypertension and reduced glomerular filtration rate [12]. As it is usually impractical to confirm by biopsy, the disease is typically diagnosed and monitored by means of subject albuminuria levels [12]. Although a continuous variable by nature, albuminuria may be categorised into three stages, with the lowest stage, A1 (normoalbuminuria), implying an absence of clinically significant nephropathy and stages A2 (microalbuminuria) and A3 (macroalbuminuria) implying the presence of clinically significant nephropathy [12]. Within 15 years of diagnosis of T2DM, approximately 35% of patients will have developed clinically significant albuminuria (stage ≥A2) [13].

T2DM and albuminuria have both been independently associated with an increased risk of mortality from cardiovascular disease [14, 15] and cancer [16, 17]. Shared dependence between the distribution of each of these outcomes and both T2DM and albuminuria renders the non-informative censoring assumption unlikely to be valid, as the distributions of censoring events (primarily driven by competing mortality) and primary outcome events will both be associated with the severity of albuminuria and T2DM. Hence, significant bias may be present in measures of risk estimated using standard survival analysis methods in populations with T2DM and albuminuria.

Using the impact of albuminuria on competing mortality in T2DM as a case study, we set out to evaluate the effect of implementing competing-risks-adjusted methods on measures of absolute risk and measures of association.

## Methods

### Data source

The Clinical Practice Research Datalink (CPRD) is the world’s largest database of anonymised primary care data, spanning 674 practices in the UK [18]. The database contains the records of 11.3 million patients, 4.4 million of which were alive and under observation on 2 July 2013, representing 6.9% of the UK population [18]. Data on patient diagnostic codes, prescriptions, referrals, and laboratory and test data are automatically collected from each contributing practice. The protocol for this research was approved by the Independent Scientific Advisory Committee (ISAC) of the Medicines and Healthcare Products Regulatory Agency (protocol number 15_011A), and the approved protocol was made available to the journal and reviewers during peer review. Ethical approval for observational research using the CPRD with approval from ISAC has been granted by a National Research Ethics Service committee (Trent MultiResearch Ethics Committee, REC reference number 05/MRE04/87).

### Study population

Patient eligibility was defined using only data uploaded by GP practices after the date they were classified as up-to-standard. Patients were required to be aged 35 years or over with T2DM on the study start date, have gender unambiguously recorded, have CPRD records that could be linked to Office for National Statistics data and have at least 2 years or uninterrupted follow-up data from their current registered general practice before the study start date for the evaluation of baseline risk factors. The presence of T2DM was established using a combination of medical codes, product codes and patient age.

Patients were excluded whose records contained medical codes for other forms of diabetes, polycystic ovary syndrome, total pancreatectomy or pancreatic or renal transplant, at any point prior to the study start date.

Follow-up commenced on the study start date of 1 January 2005 and terminated on the study end date of 1 January 2014. Patient follow-up ceased at the earliest event of the study end date, the date of patient mortality, the date of last data collection from the patient’s practice, the date the patient transferred out of their current practice and the date the patient underwent total pancreatectomy, or pancreatic or renal transplant.

### Main exposure variable

Albuminuria status was defined using definitions present in the 2013 Kidney Disease Improving Global Outcomes guidelines [12]. Within this, albuminuria stage A1 was classified as normoalbuminuria, while albuminuria stages A2 or A3 were classified as albuminuria. The presence or absence of albuminuria in CPRD patient records was established using a combination of Read codes and test results.

### Study adjustment variables

Adjustment variable data were extracted from CPRD on patient: age, gender, body mass index (BMI), smoking status, systolic blood pressure (SBP), glycated haemoglobin (HbA_1c_) and total-to-high-density-lipoprotein (Total:HDL) cholesterol ratio.

### Outcomes

Outcome data was established using International Classification of Diseases version 10 (ICD-10) codes for the ‘underlying cause’ of death listed on UK Office for National Statistics death certificates. Within these, cardiovascular mortality was defined as death with ICD-10 codes I10-I79, cancer mortality was defined as death with ICD-10 codes C00-C97 and other mortality as death with ICD-10 codes other than those listed for cardiovascular or cancer mortality.

### Summary analysis

Comparisons between patient albuminuria status and baseline patient characteristics were performed using unpaired two-tailed *t* tests [19] for continuous outcomes and Fisher’s exact test [20] for categorical outcomes. All *p* values were adjusted using Bonferroni’s method [21–23] to correct for multiple comparisons.

Log-rank tests [24–27] were used to appraise the equality of the survival functions for each albuminuria status towards each outcome, while Gray’s K-sample test [28] was used to appraise the equality of the cumulative incidence functions for each albuminuria status towards each outcome.

### Estimators and models

#### Absolute risk

The complement of the Kaplan-Meier estimate of survival probability (1-KM) [1], herein referred to as the ‘Kaplan-Meier method’, estimates marginal risk: the cumulative risk by time *t* is an estimate of the risk of failure from a specific cause in the hypothetical case that all other causes of failure are absent. Briefly, the Kaplan-Meier method achieves this by removing from the at-risk set, at any instant, individuals who have previously experienced either the event of interest or any censoring event that prevents observation of the event of interest [4]. Competing outcomes are not considered except as a form of censoring.

The cumulative incidence competing risk (CICR) [3] method estimates absolute risk accounting for competing risks: the cumulative risk by time *t* is an estimate of the risk of failure from a specific cause, acknowledging that the absolute risk of the event is lowered by the presence of other competing risks. Individuals are removed from the at-risk set, at any instant, only if they have previously experienced the primary event or any censoring events that are explicitly assumed to be non-informative but retained in the at-risk set if they have experienced a competing outcome [4].

#### Measures of association

The Cox proportional hazards model [2] estimates cause-specific relative hazard: the ratio of the instantaneous risk in at-risk individuals with one exposure status to the instantaneous risk in at-risk individuals with another exposure status. To obtain its estimate of the cause-specific hazard ratio, the Cox proportional hazards model assumes all individuals under observation experience either the primary outcome or non-informative censoring [29]. The unstratified Lunn-McNeil competing risk model [6], herein referred to as the ‘Lunn-McNeil model’, also estimates the cause-specific hazard ratio but allows for the modelling of non-informative censoring mechanisms as competing outcomes, while assuming a common baseline hazard distribution between outcomes. In these estimates of cause-specific relative hazard, individuals are considered to be at-risk at any instant if they have not yet experienced any of the study outcomes. When using the Lunn-McNeil model to evaluate cardiovascular mortality, cancer mortality and other mortality were modelled as separate competing outcomes. When using the Lunn-McNeil model to evaluate cancer mortality, cardiovascular mortality and other mortality were modelled as separate competing outcomes. The Fine-Gray competing risk model [7] estimates the subdistribution hazard ratio: the ratio of the instantaneous risks defined as above, except that individuals are considered to be at-risk if they have not yet experienced the primary outcome [29]. Individuals are retained in the at-risk set if they have previously experienced competing risk events, analogously to the CICR method for absolute risk. When using the Fine-Gray model to evaluate cardiovascular mortality, non-cardiovascular mortality was modelled as a single competing outcome. When using the Fine-Gray model to evaluate cancer mortality, non-cancer mortality was modelled as a single competing outcome.

When competing risks are present, the different risk sets employed by cause-specific hazard models (like the Cox-PH or Lunn-McNeil model) and subdistribution hazard models (like the Fine-Gray model) give rise to different measures of association. The cause-specific hazard ratio may be thought of as a measure of ‘aetiological association’, i.e. best suited to quantifying causal relationships. Conversely, the subdistribution hazard ratio may be thought of as a measure of ‘prognostic association’, i.e. best suited to quantifying predictive relationships [30].

The proportional cause-specific hazard assumptions of the Cox-PH and Lunn-McNeil models are assessed using Schoenfeld residuals [31]. Schoenfeld-type residuals [32] are used to assess the proportional subdistribution hazard assumption of the Fine-Gray models.

### Sensitivity analyses

Sensitivity analyses were conducted to assess the robustness of all risk estimates to the misclassification of cause of mortality on patient death certificates. Within these analyses, mortality was reclassified as being attributable to either cardiovascular disease or cancer if the competing cause was listed as a contributing factor. The potential misspecification of the study primary exposure was assessed by re-assigning patient baseline albuminuria status using only read codes, numerical test values or categorical test values. Additionally, the robustness of estimates of absolute risk was evaluated through the use of an alternate cumulative risk estimator (the Nelson-Aalen estimator), while the robustness of estimates of relative risk was evaluated through the inclusion of time-interaction terms to correct for violations of the proportionality assumption, and the use of alternate parameterisations of the Lunn-McNeil model, in which competing mortality was restructured into a single outcome, representing all mortality not attributable to the primary outcome.

## Results

### Summary analysis

Of the 86,962 patients eligible for inclusion, it was possible to ascertain the baseline presence/absence of albuminuria in 54,801; 42,662 without albuminuria and 12,139 with albuminuria. Patients with albuminuria at baseline were older, had higher systolic blood pressure, had worse glycaemic control and were more likely to be current or ex-smokers. No significant associations were found between baseline albuminuria and gender, body mass index or total-to-high-density-lipoprotein cholesterol ratio. Over the course of 9 years of follow-up (median 7.7 years), 14,201 patients died: 9558 (67.3%) with normoalbuminuria at baseline and 4643 (32.7%) with albuminuria at baseline. Of these 14,201 patients, 5574 (39.3%) died from cardiovascular causes, 3455 (24.3%) died from cancer and 5172 (36.4%) died from other causes. Further detail pertaining to the entire study cohort is presented in Table 1.

**Table 1:** Baseline characteristics of the study cohort, stratified by albuminuria status. Data represent means ± SD unless a percentage (%) is stated

Baseline variable	Everyone (*n* = 54,801)	Normoalbuminuria (*n* = 42,662)	Albuminuria (*n* = 12,139)	*p* value*
Men, *n* (%)	30,164 (55.0%)	23,611 (55.3%)	6553 (54.0%)	0.081
Age (years)	67.4 ± 11.9	66.8 ± 11.8	69.4 ± 12.2	< 0.001
BMI (kg/m^2^)	29.7 ± 5.9	29.7 ± 5.9	29.8 ± 6.2	0.135
SBP (mmHg)	138.4 ± 16.9	137.7 ± 16.4	140.8 ± 18.4	< 0.001
Total: HDL cholesterol ratio	3.8 ± 1.2	3.8 ± 1.2	3.8 ± 1.2	0.423
HbA_1c_ (mmol/mol)	56.9 ± 15.3	56.1 ± 14.7	59.6 ± 17.0	< 0.001
Never smoked, *n* (%)	13,308 (24.3%)	10,499 (24.6%)	2809 (23.1%)	< 0.001
Ex-smokers, *n* (%)	28,755 (52.5%)	22,604 (53.0%)	6151 (50.7%)	
Current smokers, *n* (%)	12,738 (23.2%)	9559 (22.4%)	3179 (26.2%)	

*Adjusted for multiple comparisons using Bonferroni’s method

Among 5574 patients who died from cardiovascular causes, 3661 (65.7%) had normoalbuminuria at baseline and 1913 (34.3%) had albuminuria at baseline. Among 3455 patients who died from cancer, 2564 (74.2%) had normoalbuminuria at baseline and 891 (25.8%) had albuminuria at baseline. Among 5172 patients who died from causes other than cardiovascular disease or cancer, 3330 (64.4%) had normoalbuminuria at baseline and 1842 (35.6%) had albuminuria at baseline. Statistically significant differences (*p* < 0.001) were found between the survival functions and cumulative incidence functions of the albuminuria strata for both cardiovascular and cancer mortality.

### Measures of absolute risk

**Fig. 1:**
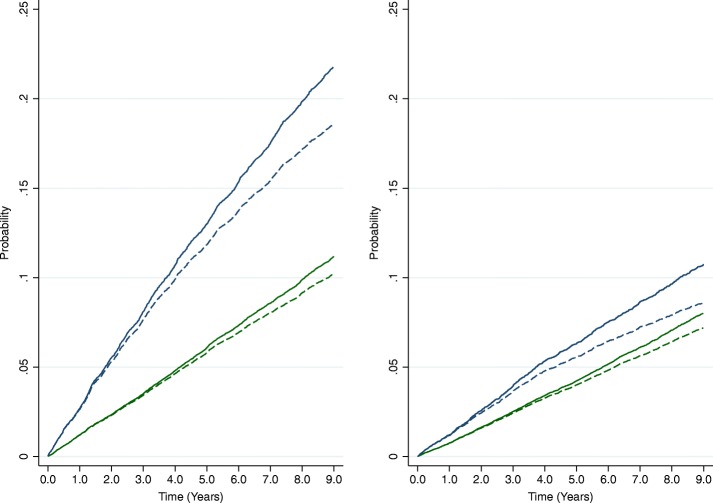
Cumulative risk estimates for cardiovascular (left) and cancer (right) mortality. Kaplan-Meier, solid lines; cumulative incidence competing risk, dashed lines; normoalbuminuria, green lines; albuminuria, blue lines

Estimates from Kaplan-Meier and CICR estimators were similar for the first 2 to 3 years but progressively diverged with the passage of time (Fig. 1). For both outcomes in patients with and without albuminuria, cumulative risk estimates from Kaplan-Meier estimators produced higher 9-year risk estimates than cumulative incidence estimates from CICR estimators (Table 2).

**Table 2:** Nine-year absolute risk estimates (and 95%CIs) for cardiovascular and cancer mortality from Kaplan-Meier and cumulative incidence competing risk estimators

Albuminuria status	Cardiovascular mortality risk (%)		Cancer mortality risk (%)	
	Kaplan-Meier	CICR	Kaplan-Meier	CICR
Normoalbuminuria	11.1 (10.8–11.5)	10.2 (9.9–10.5)	8.0 (7.7–8.3)	7.2 (6.9–7.5)
Albuminuria	21.8 (20.9–22.7)	18.5 (17.8–19.3)	10.7 (10.0–11.5)	8.6 (8.1–9.2)

### Measures of relative risk

Albuminuria was found to be significantly associated with the cause-specific hazards and subdistribution hazards of cardiovascular and cancer mortality in both univariable and multivariable analyses (Table 3, Additional file 1: Table S1 and Additional file 2: Table S2). For all types of proportional hazards models, the coefficient for the albuminuria variable (β_albuminuria_) was larger in variable-unadjusted models and larger for the outcome of cardiovascular mortality. For a given outcome, model estimates from the Cox-PH and Lunn-McNeil cause-specific hazard models were similar, whereas model estimates from the subdistribution hazard Fine-Gray model were lower. Regardless of these trends, model estimates within respective outcomes did not differ to a degree that would have resulted in drastically different clinical implications had the incorrect measure of risk been modelled.

**Table 3:** Estimates for the effect of albuminuria status on cardiovascular and cancer mortality from univariable and multivariable proportional hazards models. Adjusted for age, gender, BMI, smoking status, SBP, HbA_1c_ and Total:HDL cholesterol ratio. β_albuminuria_ refers to the model coefficient for the albuminuria variable

Model	Cardiovascular mortality β_albuminuria_ (95%CI)		Cancer mortality β_albuminuria_ (95%CI)	
	Univariable	Multivariable*	Univariable	Multivariable*
Cox-PH	0.755 (0.700–0.811)	0.557 (0.491–0.623)	0.349 (0.273–0.425)	0.237 (0.148–0.326)
Lunn-McNeil	0.759 (0.703–0.814)	0.561 (0.494–0.628)	0.351 (0.275–0.428)	0.244 (0.154–0.333)
Fine-Gray	0.670 (0.615–0.725)	0.456 (0.389–0.523)	0.223 (0.147–0.300)	0.102 (0.012–0.192)

*Adjusted for age, gender, BMI, smoking status, SBP, HbA_1c_ and Total: HDL cholesterol ratio

### Sensitivity analyses

All analyses were robust to the use of alternative outcome structures in the Lunn-McNeil model, the use of different components of CPRD to define albuminuria and the imputation of missing patient albuminuria status as normoalbuminuria. Absolute risk estimates were sensitive to the reclassification of mortality, while relative risk estimates were sensitive to the inclusion of time interactions. In all analyses, where values of cumulative risk or β_albuminuria_ were not similar to those described by the primary analyses, the patterns of association described between estimators and models remained unchanged.

## Discussion

### Key results

Numerical measures of absolute risk were consistently higher by the Kaplan-Meier estimator than by the CICR estimator, especially in patients with albuminuria. This was true for both outcomes studied, cardiovascular and cancer mortality.

Numerical measures of association between albuminuria and each outcome varied according to the approach taken to competing risks. Competing risks adjusted estimates of cause-specific hazard ratios, from the Lunn-McNeil model, were similar to conventional estimates of cause-specific hazard ratios from the Cox model. Competing risks adjusted estimates of subdistribution hazard ratios, from the Fine-Gray model, were numerically smaller than estimates of cause-specific hazard ratios, reflecting the different interpretation. In all methods, the associations between albuminuria and outcomes were positive and statistically significant.

### Strengths and limitations

All primary analyses carried out within this study were the result of protocol-driven research, approved by ISAC (protocol 15_011A). Strengths of the CPRD dataset include its size and its representativeness of the UK [18]. Limitations of the data source include missing data in the electronic health record, particularly for covariates such as body mass index which are not universally measured. The Office for National Statistics records of death certification provides a highly complete record of mortality. Mortality cannot always be unambiguously attributed to a single cause, but in a sensitivity analysis (not shown, see Feakins, DPhil thesis [33]), we found that our numerical comparisons and overall conclusions were little altered by different approaches to death certificates with both cardiovascular and cancer codes.

This is a single case study of competing risk methodology, limited to methods identified as being commonly used in the literature and readily implementable in statistical packages. Thus, the results described may not mirror the findings in other clinical areas, or from different models.

### Relationship to the literature

Our findings for absolute risk estimates are consistent with those from studies of cancer progression in patients with head or neck cancer [4], cancer incidence in patients with chronic kidney disease [34], cardiovascular mortality in patients who underwent renal replacement therapy [3], renal transplantation in patients on dialysis [30] and coronary artery bypass in patients who underwent cardiac catheterisation [35]. It also corroborated the results of the initial simulation work performed by Gooley et al. [4] in the derivation of the CICR estimator [4]. The results of this study further add to the evidence of bias in the Kaplan-Meier estimator by demonstrating its presence in the novel clinical areas of cardiovascular and cancer mortality in patients with T2DM.

Several authors have previously compared Fine-Gray to Cox models [7, 29, 30, 36–44]. However, only four studies were identified in the literature in which the effect of risk factors were explicitly modelled on both the primary outcome and competing outcomes using cause-specific hazard and subdistribution hazard models [29, 30, 42, 44]. Of these, only a single study was identified in which the model comparisons mirrored those of this study [29]. Our findings confirm the observation of Lau et al. [29] that the subdistribution hazard ratio is attenuated compared to the Cox cause-specific hazard ratio when the exposure acts in the same direction on both the primary and competing outcomes (although this was not guaranteed for our study as we analysed the main competing outcome, as opposed to all competing outcomes). One previous paper included all three of the Cox, Lunn-McNeil and Fine-Gray models. This was a study of risk factors for end-stage renal disease, with death from all non-renal causes as the competing outcome [44]. Differences between the three methods were more pronounced than ours for the primary outcome, with even the Lunn-McNeil estimates of association attenuated compared to the Cox estimates. Some Fine-Gray estimates not just attenuated but reversed in direction compared to the Cox and Lunn-McNeil estimates (Table 4). The latter effect illustrates how strongly an association between the exposure and a prevalent competing outcome can affect subdistribution hazard ratios for the primary outcome: the effect of age on overall, non-renal mortality is so strong that (in the Fine-Gray model of competing risks) it swamps the positive but weaker association of age with end-stage renal disease. Compared to that paper, our case study has a more balanced ratio of primary to competing outcomes, which may explain the differences. However, it is not clear how or whether the previous paper verified the proportionality assumption, which we assessed for both cause-specific hazards and subdistribution hazards (Additional files 3, 4, 5, 6 and 7).

**Table 4:** Comparison between the results of this study and the study by Lim et al.

Study	Outcome	Risk factor	Cox ln(HR) and 95% CI	Lunn-McNeil ln(HR) and 95% CI	Fine-Gray ln(SHR) and 95% CI
This paper	Cardiovascular mortality	Albuminuria	0.557 (0.491, 0.623)	0.561 (0.494, 0.628)	0.456 (0.389, 0.523)
This paper	Cancer mortality	Albuminuria	0.237 (0.148, 0.326)	0.244 (0.154, 0.333)	0.102 (0.012, 0.192)
Lim et al. 2010	ESRD	Male	0.414 (0.136, 0.692)	0.333 (0.055, 0.610)	0.280 (0.006, 0.555)
Lim et al. 2010	ESRD	40 < age < 60	0.139 (− 0.164, 0.441)	0.075 (− 0.227, 0.376)	− 0.080 (− 0.378, 0.218)
Lim et al. 2010	ESRD	Age ≥ 60	0.339 (− 0.115, 0.795)	0.004 (− 0.445, 0.454)	− 0.635 (− 1.094, − 0.177)
Lim et al. 2010	Death without ESRD	Male	0.320 (0.218, 0.422)	0.336 (0.234, 0.438)	0.307 (0.204, 0.404)
Lim et al. 2010	Death without ESRD	40 < age < 60	0.986 (0.818, 1.150)	0.996 (0.829, 1.164)	0.975 (0.810, 1.140)
Lim et al. 2010	Death without ESRD	Age ≥ 60	2.325 (2.158, 2.492)	2.373 (2.206, 2.562)	2.299 (2.135, 2.463)

We have not addressed here the stratified Lunn-McNeil model, because it is known to give near-identical results to the Cox model [44]. A previous study compared stratified Lunn-McNeil to both Cox and Fine-Gray models; results from all three were similar [37]. Nor have we considered the less widely used models for competing risks such as the pseudovalues approach [45], direct binomial regression [46], mixture models [29, 47], or Bayesian methods [48]; however, an excellent comparison of the various methods available can be found in Haller et al. [41].

## Conclusions

Studies of absolute risk should use methods that adjust for competing risks to avoid over-stating risk, such as the CICR estimator. Studies of relative risk should consider carefully which measure of association is most appropriate for the research question. Cause-specific hazard ratios will be appropriate when the aim of the study is to establish a potentially causal relationship between an exposure and an outcome. Clinical trials, for example, are primarily interested in the relationship between allocated treatment group and the primary outcome. In these cases where cause-specific hazard ratios are appropriate, we find, in common with previous authors, little difference between estimates from the Cox model and from the Lunn-McNeil competing risk model. Subdistribution hazard ratios will be appropriate when the aim of the study is to quantify burden of disease, for example, in combination with a CICR estimate to produce a prognostic model. As noted above, subdistribution hazard ratios have the property that a beneficial effect of the exposure on a competing outcome may appear as a harmful effect on the outcome of interest, or vice versa.

Our findings, the previous literature and common sense confirm that researchers should consider competing risks when selecting statistical analysis methods. The widely used Kaplan-Meier method arguably over-estimates absolute risk, sometimes substantially, and though the widely used Cox proportional hazards method for relative risk will often be a reasonable approximation, users should at least be making an informed choice to use cause-specific rather than subdistribution hazard methods.

## Additional files

Additional file 1: **Table S1.** Adjusted estimates for the effect of baseline risk factors on cardiovascular mortality from Cox-PH, Lunn-McNeil and Fine-Gray Models. (DOCX 15 kb)

Additional file 2: **Table S2.** Adjusted estimates for the effect of baseline risk factors on cancer mortality from Cox-PH, Lunn-McNeil and Fine-Gray Models. (DOCX 15 kb)

Additional file 3: **Figure S1.** Proportional cause-specific hazards assessment cardiovascular mortality. (PDF 1874 kb)

Additional file 4: **Figure S2.** Proportional cause-specific hazards assessment for cancer mortality. (PDF 1233 kb)

Additional file 5: **Figure S3.** Proportional cause-specific hazards assessment for other mortality. (PDF 1695 kb)

Additional file 6: **Figure S4.** Proportional subdistribution hazards assessment for cardiovascular mortality. (PDF 1100 kb)

Additional file 7: **Figure S5.** Proportional subdistribution hazards assessment for cancer mortality. (PDF 865 kb)

## Abbreviations

BMI: Body mass index
CI: Confidence interval
CICR: Cumulative incidence competing risk
CPRD: Clinical Practice Research Datalink
CSH: Cause-specific hazard
GP: General practitioner
HbA_1c_: Glycated haemoglobin
ICD: International Classification of Diseases
ISAC: Independent Scientific Advisory Committee
SBP: Systolic blood pressure
SDH: Subdistribution hazard
T2DM: Type 2 diabetes mellitus
Total:HDL: Total-to-high-density lipoprotein
