# Supplementary material for: Standard and competing risk analysis of the effect of albuminuria on cardiovascular and cancer mortality in patients with type 2 diabetes mellitus
Source: Diagn Progn Res. 2018 Jul 23;2:13. doi: 10.1186/s41512-018-0035-4 (PMC6460530; doi:10.1186/s41512-018-0035-4)
Supplement: Supplementary file 1 — Table S1. Adjusted estimates for the effect of baseline risk factors on cardiovascular mortality from Cox-PH, Lunn-McNeil and Fine-Gray Models. (DOCX 15 kb) [file 41512_2018_35_MOESM1_ESM.docx]

Table S1 - Adjusted estimates for the effect of baseline risk factors on cardiovascular mortality from Cox-PH, Lunn-McNeil and Fine-Gray Models.

| **Variable** | **Cox-PH Model** | | **Lunn-McNeil Model** | | **Fine-Gray Model** | |
| --- | --- | --- | --- | --- | --- | --- |
|  | **β_albuminuria_** | **P-Value** | **β_albuminuria_** | **P-Value** | **β_albuminuria_** | **P-Value** |
| Albuminuria Status | 0.557  (0.491 - 0.623) | <0.001 | 0.561  (0.494 - 0.628) | <0.001 | 0.456  (0.388 - 0.523) | <0.001 |
| Male Gender | 0.309  (0.244 - 0.373) | <0.001 | 0.310  (0.245 - 0.374) | <0.001 | 0.266  (0.201 - 0.331) | <0.001 |
| Age | 0.104  (0.100 - 0.108) | <0.001 | 0.105  (0.101 - 0.109) | <0.001 | 0.088  (0.085 - 0.092) | <0.001 |
| BMI | 0.016  (0.010 - 0.023) | <0.001 | 0.016  (0.010 - 0.023) | <0.001 | 0.018  (0.012 - 0.024) | <0.001 |
| Total : HDL Cholesterol | 0.058  (0.031 - 0.085) | <0.001 | 0.059  (0.032 - 0.086) | <0.001 | 0.051  (0.024 - 0.077) | <0.001 |
| HbA_1c_ | 0.013  (0.011 - 0.015) | <0.001 | 0.013  (0.011 - 0.015) | <0.001 | 0.011  (0.009 - 0.013) | <0.001 |
| SBP | -0.002  (-0.004 - -0.000) | 0.014 | -0.002  (-0.004 - -0.000) | 0.024 | -0.001  (-0.003 - 0.001) | 0.509 |
| Ex-Smoker | 0.162  (0.080 - 0.245) | <0.001 | 0.164  (0.082 - 0.246) | <0.001 | 0.147  (0.065 - 0.230) | <0.001 |
| Current Smoker | 0.441  (0.344 - 0.538) | <0.001 | 0.444  (0.347 - 0.541) | <0.001 | 0.377  (0.280 - 0.474) | <0.001 |
