# Supplementary material for: Standard and competing risk analysis of the effect of albuminuria on cardiovascular and cancer mortality in patients with type 2 diabetes mellitus
Source: Diagn Progn Res. 2018 Jul 23;2:13. doi: 10.1186/s41512-018-0035-4 (PMC6460530; doi:10.1186/s41512-018-0035-4)
Supplement: Supplementary file 2 — Table S2. Adjusted estimates for the effect of baseline risk factors on cancer mortality from Cox-PH, Lunn-McNeil and Fine-Gray Models. (DOCX 15 kb) [file 41512_2018_35_MOESM2_ESM.docx]

Table S2 - Adjusted estimates for the effect of baseline risk factors on cancer mortality from Cox-PH, Lunn-McNeil and Fine-Gray Models.

| **Variable** | **Cox-PH Model** | | **Lunn-McNeil Model** | | **Fine-Gray Model** | |
| --- | --- | --- | --- | --- | --- | --- |
|  | **β_albuminuria_** | **P-Value** | **β_albuminuria_** | **P-Value** | **β_albuminuria_** | **P-Value** |
| Albuminuria Status | 0.237  (0.148 - 0.326) | <0.001 | 0.244  (0.154 - 0.333) | <0.001 | 0.102  (0.012 - 0.192) | 0.026 |
| Male Gender | 0.364  (0.281 - 0.446) | <0.001 | 0.365  (0.282 - 0.448) | <0.001 | 0.337  (0.255 - 0.420) | <0.001 |
| Age | 0.069  (0.065 - 0.073) | <0.001 | 0.071  (0.066 - 0.075) | <0.001 | 0.053  (0.049 - 0.057) | <0.001 |
| BMI | 0.008  (-0.000 - 0.015) | 0.056 | 0.008  (-0.000 - 0.015) | 0.058 | 0.009  (0.001 - 0.016) | 0.019 |
| Total : HDL Cholesterol | 0.020  (-0.017 - 0.057) | 0.287 | 0.021  (-0.016 - 0.058) | 0.259 | 0.008  (-0.028 - 0.045) | 0.653 |
| HbA_1c_ | 0.001  (-0.002 - 0.004) | 0.462 | 0.001  (-0.002 - 0.004) | 0.411 | -0.001  (-0.004 - 0.002) | 0.502 |
| SBP | -0.004  (-0.006 - -0.001) | 0.003 | -0.004  (-0.006 - -0.001) | 0.002 | -0.002  (-0.004 - 0.000) | 0.073 |
| Ex-Smoker | 0.350  (0.239 - 0.461) | <0.001 | 0.352  (0.241 - 0.463) | <0.001 | 0.336  (0.225 - 0.447) | <0.001 |
| Current Smoker | 0.791  (0.669 - 0.913) | <0.001 | 0.797  (0.675 - 0.919) | <0.001 | 0.735  (0.613 - 0.857) | <0.001 |
