# Supplementary figures and images for: Standard and competing risk analysis of the effect of albuminuria on cardiovascular and cancer mortality in patients with type 2 diabetes mellitus
Source: Diagn Progn Res. 2018 Jul 23;2:13. doi: 10.1186/s41512-018-0035-4 (PMC6460530; doi:10.1186/s41512-018-0035-4)

Scaled Schoenfeld Residuals

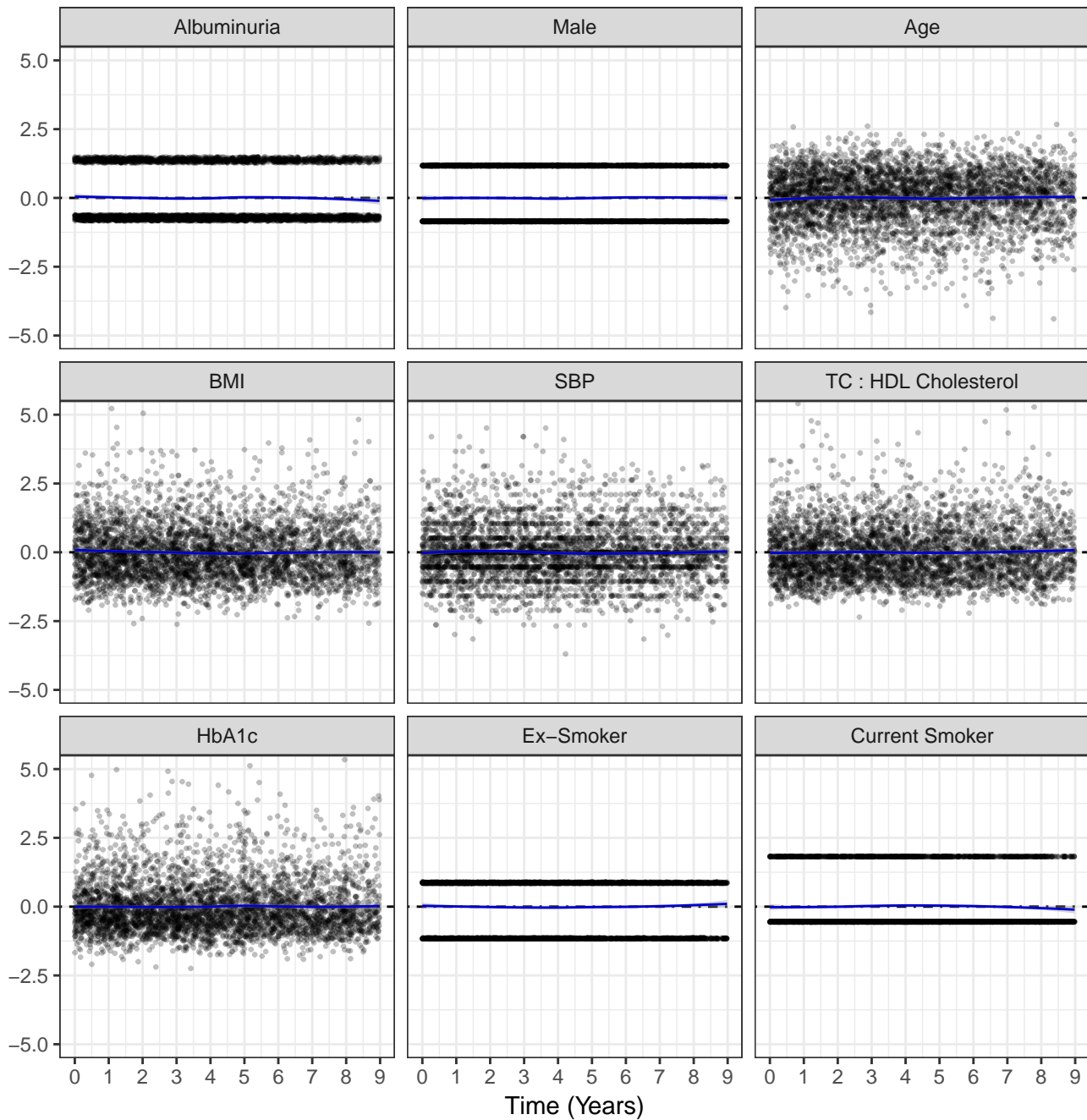

Supplement: Supplementary file 3 — Figure S1. Proportional cause-specific hazards assessment cardiovascular mortality. (PDF 1874 kb) [file 41512_2018_35_MOESM3_ESM.pdf]

Scaled Schoenfeld Residuals

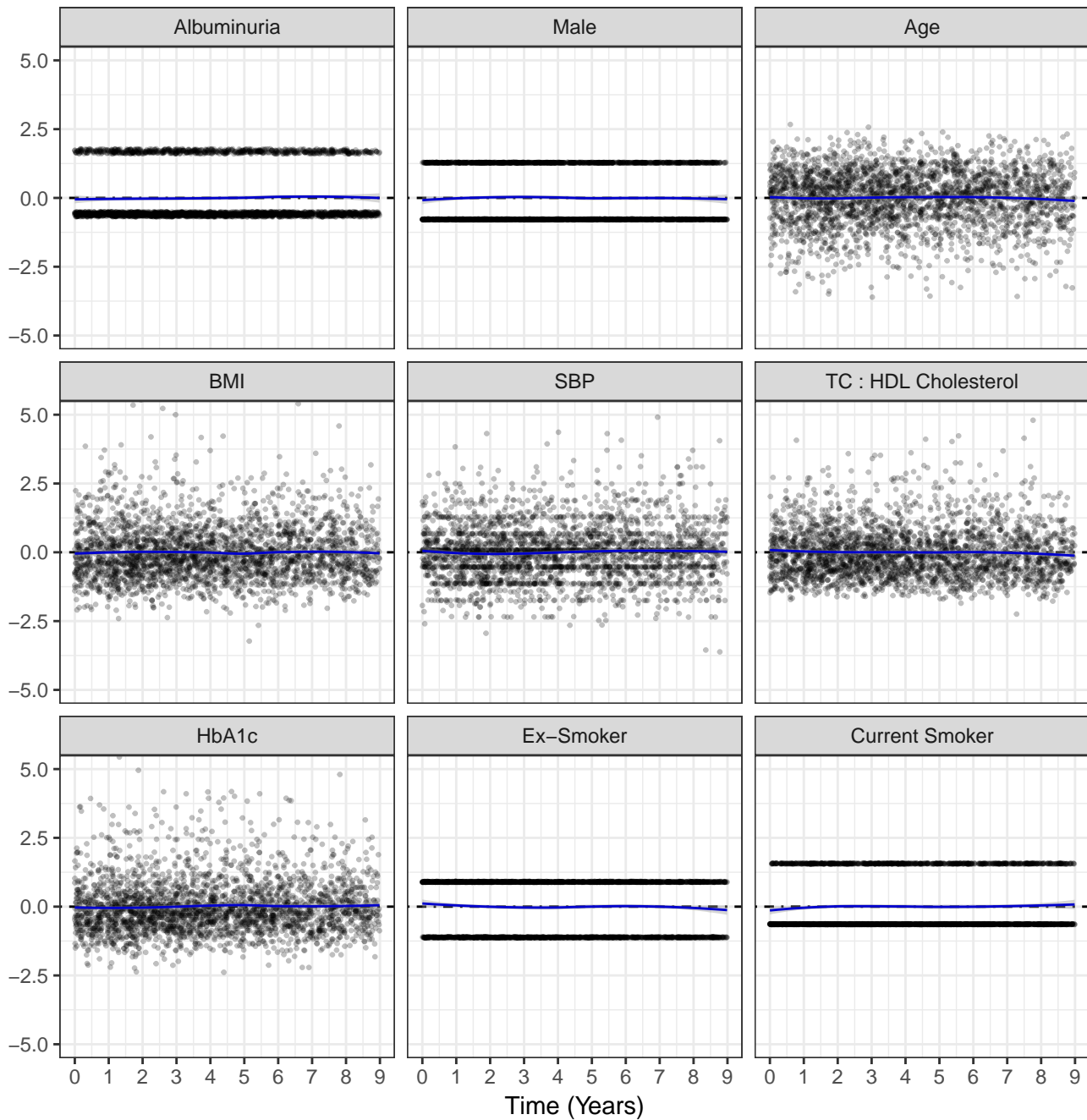

Supplement: Supplementary file 4 — Figure S2. Proportional cause-specific hazards assessment for cancer mortality. (PDF 1233 kb) [file 41512_2018_35_MOESM4_ESM.pdf]

Scaled Schoenfeld Residuals

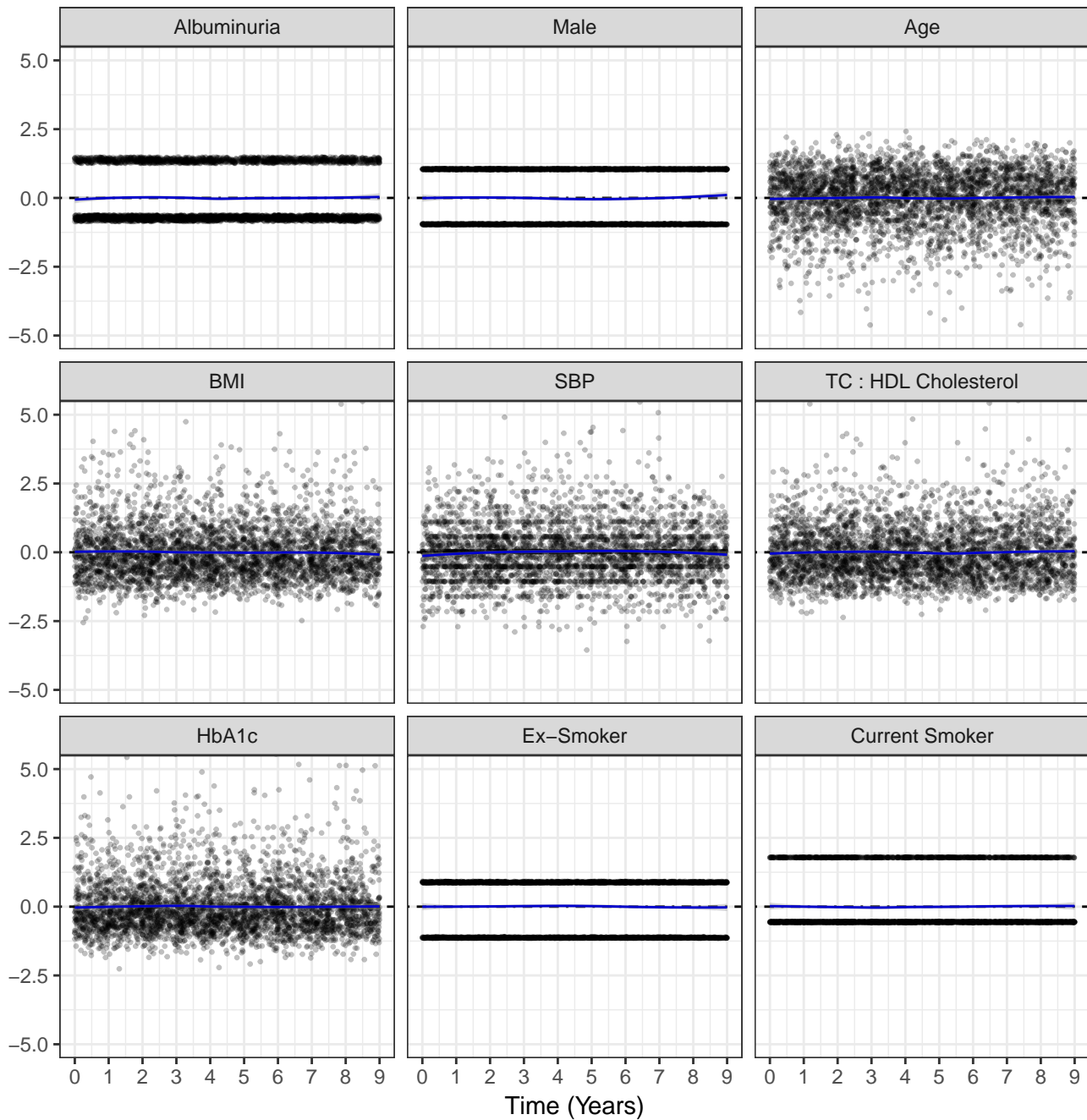

Supplement: Supplementary file 5 — Figure S3. Proportional cause-specific hazards assessment for other mortality. (PDF 1695 kb) [file 41512_2018_35_MOESM5_ESM.pdf]

Scaled Schoenfeld-Type Residuals

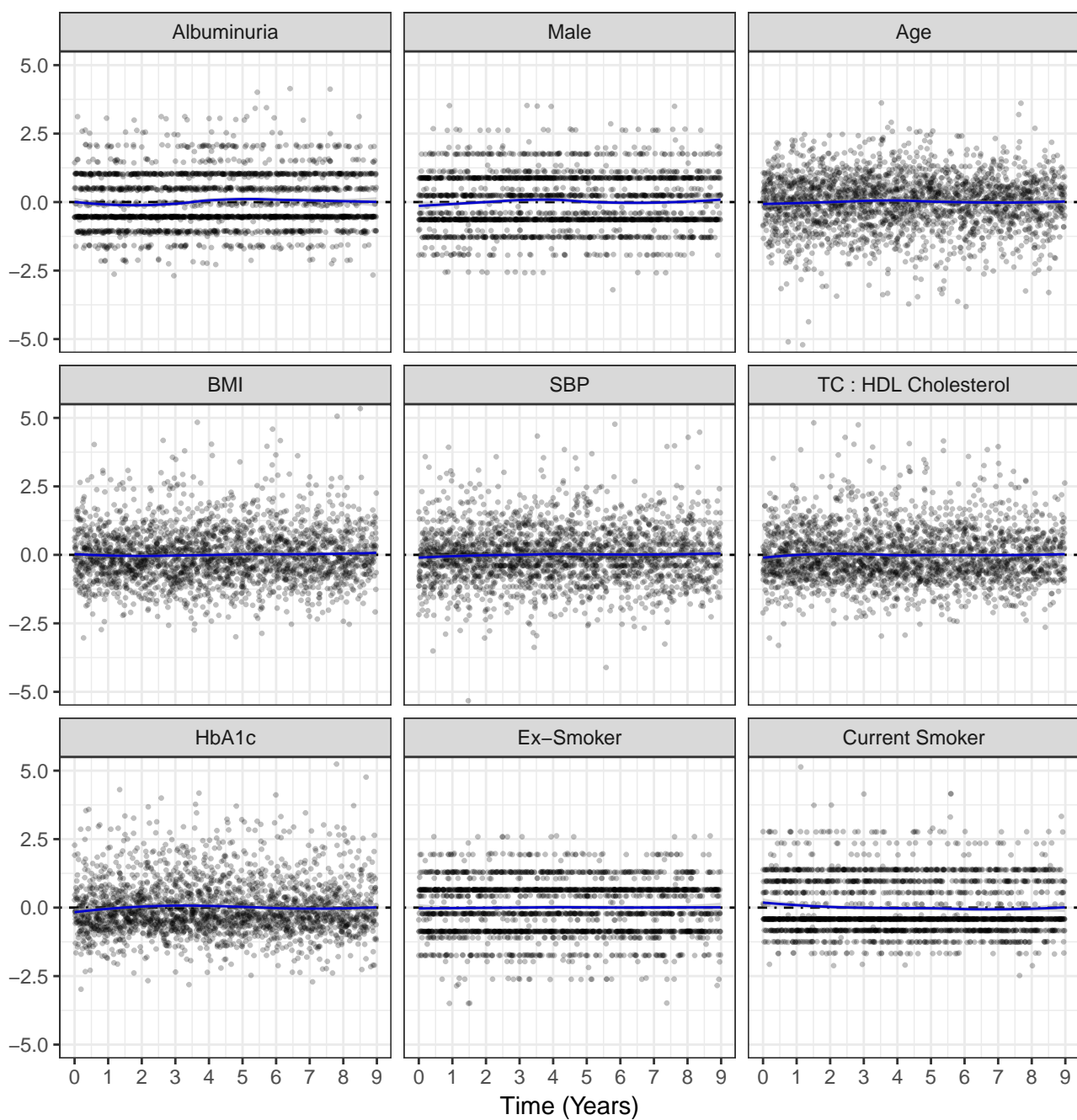

Supplement: Supplementary file 6 — Figure S4. Proportional subdistribution hazards assessment for cardiovascular mortality. (PDF 1100 kb) [file 41512_2018_35_MOESM6_ESM.pdf]

Scaled Schoenfeld-Type Residuals

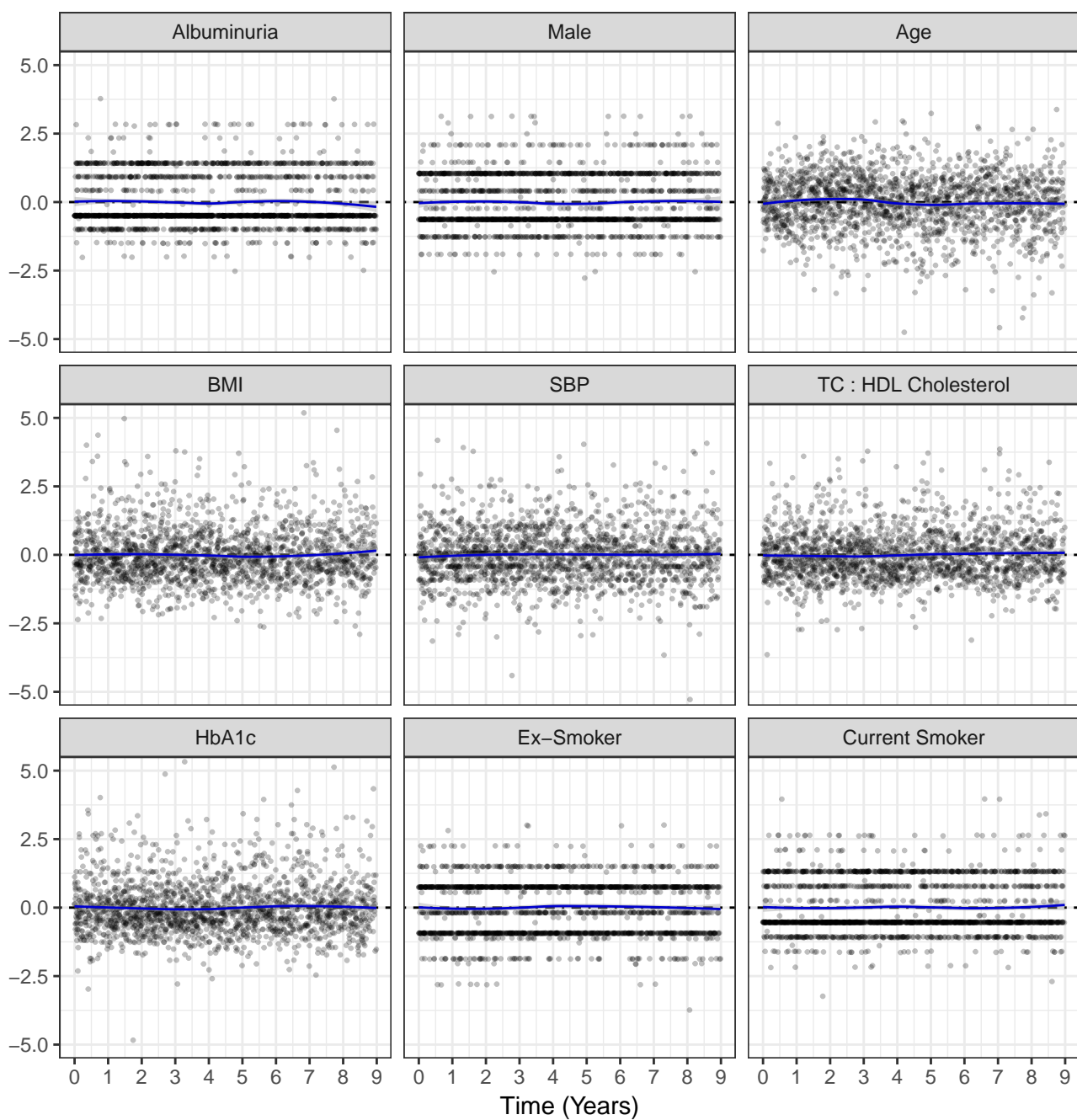

Supplement: Supplementary file 7 — Figure S5. Proportional subdistribution hazards assessment for cancer mortality. (PDF 865 kb) [file 41512_2018_35_MOESM7_ESM.pdf]
